# Symptom clusters and their influence on prognosis using EORTC QLQ-C15-PAL scores in terminally ill patients with cancer

**DOI:** 10.1007/s00520-021-06380-w

**Published:** 2021-07-09

**Authors:** Nanako Koyama, Chikako Matsumura, Yuuna Tahara, Morito Sako, Hideo Kurosawa, Takehisa Nomura, Yuki Eguchi, Kazuki Ohba, Yoshitaka Yano

**Affiliations:** 1 Education and Research Center for Clinical Pharmacy, Kyoto Pharmaceutical University, 5 Nakauchi-choYamashina-ku, MisasagiKyoto, 607-8414 Japan; 2 Department of Pharmacy, Tachibana Medical Corporation Higashisumiyoshi-Morimoto Hospital, Osaka, Japan; 3 Palliative Care Unit, Tachibana Medical Corporation Higashisumiyoshi-Morimoto Hospital, Osaka, Japan; 4 Department of Palliative Care, Tachibana Medical Corporation Higashisumiyoshi-Morimoto Hospital, Osaka, Japan

**Keywords:** Terminally ill cancer patient, Symptom cluster, EORTC QLQ-C15-PAL score, Palliative Performance Scale, Survival prediction

## Abstract

**Purpose:**

The aims of the present study were to investigate the symptom clusters in terminally ill patients with cancer using the European Organization for Research and Treatment of Cancer Quality of Life Questionnaire Core 15 Palliative Care (EORTC QLQ-C15-PAL), and to examine whether these symptom clusters influenced prognosis.

**Methods:**

We analyzed data from 130 cancer patients hospitalized in the palliative care unit from June 2018 to December 2019 in an observational study. Principal component analysis was used to detect symptom clusters using the scored date of 14 items in the QLQ-C15-PAL, except for overall QOL, at the time of hospitalization. The influence of the existence of these symptom clusters and Palliative Performance Scale (PPS) on survival was analyzed by Cox proportional hazards regression analysis, and survival curves were compared between the groups with or without existing corresponding symptom clusters using the log-rank test.

**Results:**

The following symptom clusters were identified: cluster 1 (pain, insomnia, emotional functioning), cluster 2 (dyspnea, appetite loss, fatigue, and nausea), and cluster 3 (physical functioning). Cronbach’s alpha values for the symptom clusters ranged from 0.72 to 0.82. An increased risk of death was significantly associated with the existence of cluster 2 and poor PPS (log-rank test, p = 0.016 and p < 0.001, respectively).

**Conclusion:**

In terminally ill patients with cancer, three symptom clusters were detected based on QLQ-C15-PAL scores. Poor PPS and the presence of symptom cluster that includes dyspnea, appetite loss, fatigue, and nausea indicated poor prognosis.

## Introduction

It is well-known that patients with advanced cancer experience various distressing symptoms as their end-of-life approaches. In particular, symptoms such as dyspnea and fatigue have been found to increase in severity compared with symptoms such as pain, nausea, anxiety, and depression [1]. Therefore, most patients with cancer seek relief from suffering at the end of their lives. Most studies regarding cancer-related symptoms in palliative care settings have focused on a single symptom, but recently, knowledge dissemination and application of symptom clusters have been proposed for palliative care [2].

Symptom clusters are defined as two or more inter-related symptoms that are present together, independent of other symptom clusters [3, 4]. Dong et al. demonstrated that patients with advanced cancer had four common symptom clusters: anxiety–depression, nausea–vomiting, nausea–appetite loss, and fatigue–dyspnea–drowsiness–pain. However, a few studies included in this review were conducted on terminally ill cancer patients [3]. Although the Edmonton Symptom Assessment System (ESAS) is frequently used as a symptom assessment tool, quality-of-life (QOL) questionnaires such as the European Organization for Research and Treatment of Cancer Quality of Life Questionnaire Core 15 Palliative Care (QLQ-C15-PAL) are also useful for assessing patient-reported outcomes (PROs) in palliative care settings [5, 6]. Therefore, we consider that it is valuable to explore symptom clusters in terminally ill patients with cancer using tools specific to palliative patient populations, such as the QLQ-C15-PAL.

For patients with advanced cancer, an accurate prediction of survival is important for clinical and personal decision-making in the last months, weeks, and days of life [7]. The Palliative Prognostic Index (PPI) is a standard tool for predicting the short-term prognosis in palliative care [8]. The PPI is composed of performance status (Palliative Performance Scale (PPS)), decline of oral intake, presence and absence of edema, dyspnea at rest, and delirium. In the parameters for PPI, individual distress symptoms such as delirium or dyspnea have been reported as independent predictors of survival in cancer patients [9]. However, these are subjective factors, and therefore it is often difficult for clinicians to use these tools for prognosis prediction unless they evaluate patients’ symptoms correctly.

We have already conducted a clinical prospective observational study to examine the possible predictors of prognosis for terminally ill patients with cancer [10]. We found that some QOL scores, such as fatigue and dyspnea using the QLQ-C15-PAL, as well as inflammatory biomarkers, such as C-reactive protein (CRP), albumin (Alb), and neutrophil-to-lymphocyte ratio (NLR), were significant prognostic factors, and we estimated the cutoff values for poor prognosis in such patients. In the same clinical study, we compared the correlations between the scores of the patients themselves and the scores of health care professionals [11]. These are often called PROs and clinician-reported outcomes (CROs), respectively. PROs and CROs showed higher correlation in pain and anorexia (appetite loss), and the degrees of exact agreement vary between fatigue (15.4%) and nausea (57.7%), which showed a negative correlation with the mean of each QLQ-C15-PAL score [11].

Throughout these studies, we found some statistically significant correlations between the items in the QLQ-C15-PAL, which means that the items are not independently scored and there are some items that behave similarly within a patient. We hypothesized that these may be symptom clusters, and it is useful to clarify the details of such symptom clusters in terminally ill patients. Ganesh et al. have investigated the symptom clusters using QLQ-C15-PAL score which is a palliative-specific patient-reported QOL assessment tool; however, the study was limited to the patients undergoing radiotherapy for advanced cancer [12]. Thus, we investigated the symptom clusters in nonselective terminally ill patients with cancer at the time of hospitalization in palliative care unit using the QLQ-C15-PAL. In addition, we examined whether these symptom clusters were related to survival prediction.

## Methods

### Design and patients

The data in this study were obtained from our prospective observational study [11], which was conducted with cancer patients newly hospitalized in the palliative care unit from June 2018 to December 2019. Among them, only the baseline data at the time of hospitalization were extracted and used for this secondary analysis. The study was conducted in accordance with the Declaration of Helsinki and Ethical Guidelines for Epidemiology Research and was approved by the Ethics Committees at the hospital on May 15, 2018, and the university with which the authors are affiliated. Patients were included in this observational study if they could answer the questionnaires during the study period. Data were collected from 130 patients’ questionnaires using the Japanese version of the QLQ-C15-PAL. The QLQ-C15-PAL is a self-report tool with 10 domains of 15 items that evaluates a patient’s QOL. In the present study, analyses were conducted using responses for 2 functional domains (physical functioning (3 items) and emotional functioning (2 items)) and 7 symptom domains (dyspnea (1 item), pain (2 items), insomnia (1 item), appetite loss (1 item), constipation (1 item), fatigue (2 items), and nausea (1 item)) on a 4-point scale (1 = not at all, 2 = a little, 3 = quite a bit, 4 = very much). Patients’ baseline data including age, sex, PPS, cancer type, presence or absence of metastasis, and inflammatory biomarkers (CRP and Alb) were collected from the medical records at the time of hospitalization. Additionally, all patients were followed up until death or discharge during the study period.

### Statistical analysis

Descriptive statistics were summarized as the prevalence and severity of the 2 functional domains (5 items) and 7 symptom domains (9 items). To detect some symptom clusters and examine whether any interrelationships exist among the functional and symptoms domains, a principal component analysis (PCA) with varimax rotation was conducted on the intensity of 14 items at the time of hospitalization in the palliative care unit. PCA transforms several observed variables into a smaller number of variables, called principal components [13]. There are some reports regarding statistical analyses of symptom clusters in cancer patients [12, 14]. In this study, we followed the analysis conditions in these previous reports, and an eigenvalue higher than 0.8 was used to select the number of significant principal components, each explaining more than 10% of the total variance and contained at least two symptoms [14]. Inter-factor correlations were examined using the values of factor loading by PCA, and the internal consistency and reliability of the derived symptom clusters were assessed with Cronbach’s alpha coefficient (< 0.5, unacceptable; ≥ 0.5, poor; ≥ 0.6, questionable; ≥ 0.7, acceptable; ≥ 0.8, good; and ≥ 0.9, excellent internal consistency) [15].

Based on the results of PCA, we examined whether the presence of these symptom clusters could be prognostic factors. At first, the scores of each item in the 2 functional domains and 7 symptom domains were re-scored as binary variables, where responses of 2, 3, or 4 corresponded to “presence” and a response of 1 corresponded to “absence.” Next, for each patient, a symptom cluster was defined as present if the patient had at least half of the items included in each symptom cluster, and the symptom cluster was defined as absent if not [16]. Moreover, we classified PPS scores into three groups, (≥ 70, 40–60, and ≤ 30) based on the results of a previous study [17]. Survival time in each patient was defined as the period from the date of admission to the palliative care unit to the date of death. Data from patients without available information regarding survival because they left the palliative care unit or remained in the palliative unit during the study period were censored. Survival data were analyzed using Cox proportional hazards regression analysis to determine whether the existence of each symptom cluster and each group of PPS scores were a significant factor in distinguishing the survival curves. The Kaplan–Meier method was applied to draw the survival curves to evaluate the influence of the existence of the symptom clusters and PPS on survival, and survival curves were compared between the groups with or without the corresponding symptom clusters using the log-rank test.

SPSS Statistics for Windows (version 22.0; IBM, Armonk, NY) and Bell Curve® for Excel Version 2.15 (Social Survey Research Information Co., Tokyo, Japan) were used to analyze the data. Statistical significance was set at p < 0.05. Missing data were excluded for each analysis.

## Results

Table 1 summarizes the patients’ backgrounds, as well as the previous study [11]. The median age was 74 years (range 32–97 years), and 71 patients (54.6%) were male. The major cancer type was lung cancer (n = 31, 23.8%), and almost all patients presented with metastasis (n = 114, 87.7%). At the end of the study period, 109 patients died, and the information for 21 patients was missing.

**Table 1:** Patient characteristics

	Number of patients (%)
Total number of patients	130
Median age (years, minimum–maximum)	74, 32–97
Sex (male/female)	71 (54.6)/59 (45.4)
PPS	
≥ 70	20 (15.4)
40–60	74 (56.9)
≤ 30	29 (22.3)
Unknown	7 (5.4)
Cancer types	
Lung	31 (23.8)
Colorectal	26 (20.0)
Pancreas	12 (9.2)
Gastric	7 (5.4)
Liver	7 (5.4)
Breast	5 (3.8)
Esophagus	5(3.8)
Ovarian	4 (3.1)
Uterine	4 (3.1)
Prostate	3 (2.3)
Other	26 (20.0)
Metastasis (no/yes)	16 (12.3)/114 (87.7)
Inflammatory biomarkers (median, range) n = 126	
CRP (mg/dL)	3.9 (< 0.1–32.1)
Alb (g/dL)	2.6 (1.2–3.9)
Death, survival time (day, median)	18 (n = 109)

*PPS*, Palliative Performance Scale; *CRP*, C-reactive protein; *Alb*, albumin

The 14 items of QLQ-C15-PAL scores except overall QOL are listed in Table 2, and the numbers of patients who claimed each of the scores in those items at the time of hospitalization in the palliative care unit are summarized. The mean score of each item, excluding nausea (Q9), ranged from 2 (a little) to 3 (quite a bit). Among all items, the highest proportion of “very much” was found in Q7 (felt weak; 44.6%), followed by Q2 (in bed; 44.4%).

**Table 2:** Summary of QLQ-C15-PAL scores in the patients at the time of hospitalization

QLQ-C15-PAL	Not at all (1)		A little (2)		Quite a bit (3)		Very much (4)		Mean ± SD	Total (N)
	N	%	N	%	N	%	N	%		
Q1: short walk	22	17.3	37	29.1	20	15.7	48	37.8	2.7 ± 1.14	127
Q2: in bed	16	12.7	20	15.9	34	27	56	44.4	3.0 ± 1.05	126
Q3: need help	34	26.8	27	21.3	23	18.1	43	33.9	2.6 ± 1.21	127
Q4: dyspnea	41	31.5	42	32.3	29	22.3	18	13.8	2.2 ± 1.03	130
Q5: pain	32	24.6	42	32.3	30	23.1	26	20	2.4 ± 1.06	130
Q6: insomnia	46	35.4	31	23.8	34	26.2	19	14.6	2.2 ± 1.08	130
Q7: felt weak	11	8.5	22	16.9	39	30	58	44.6	3.1 ± 0.97	130
Q8: appetite loss	25	19.2	33	25.4	24	18.5	48	36.9	2.7 ± 1.15	130
Q9: nausea	71	54.6	32	24.6	16	12.3	11	8.5	1.7 ± 0.97	130
Q10: been constipated	46	35.9	41	32	22	17.2	19	14.8	2.1 ± 1.06	128
Q11: been tired	16	12.4	46	35.7	26	20.2	41	31.8	2.7 ± 1.04	129
Q12: pain interference	31	24	40	31	27	20.9	31	24	2.4 ± 1.10	129
Q13: felt tense	55	42.6	33	25.6	29	22.5	12	9.3	2.0 ± 1.01	129
Q14: felt depressed	35	27.1	40	31	34	26.4	20	15.5	2.3 ± 1.03	129

*N* number of patients who claimed each symptom score, *Mean, SD* arithmetic mean and standard deviation, *Total (N)* the total number of patients from whom symptom data was obtained. These are not necessarily the same because of missing data. QLQ-C15-PAL: EORTC QLQ-C15-PAL (European Organization for Research and Treatment of Cancer Quality of Life Questionnaire Core 15 Palliative Care)

Table 3 shows the PCA results for the data at the time of hospitalization. The results of PCA suggested that the data were classified into three symptom clusters as follows: cluster 1 contained the two items of pain (Q5, Q12), emotional functioning (Q13, Q14), and insomnia (Q6); cluster 2 contained the two items of fatigue (Q7, Q11), dyspnea (Q4), appetite loss (Q8), and nausea (Q9); and cluster 3 contained the three items of physical functioning (Q1, Q2, and Q3). Constipation alone (Q10) was extracted as another cluster; however, Q10 was excluded because this cluster contained only one item.

**Table 3:** Symptoms clusters using principal component analysis at the time of hospitalization (n = 130)

Items	N (%) of patients	Inter-factor correlations	Cronbach’s alpha values	% of variance
Cluster 1 (n = 129)	92 (71.3)		0.792	20.0
Q12: pain interference		0.869		
Q5: pain		0.838		
Q13: felt tense		0.669		
Q6: insomnia		0.591		
Q14: felt depressed		0.516		
Cluster 2 (n = 129)	109 (84.5)		0.721	17.1
Q4: dyspnea		0.710		
Q8: appetite loss		0.669		
Q9: nausea		0.630		
Q7: felt weak		0.614		
Q11: been tired		0.567		
Cluster 3 (n = 125)	108 (86.4)		0.820	16.9
Q1: short walk		0.856		
Q2: in bed		0.837		
Q3: need help		0.829		

For the patients in the present study, the presence or absence of clusters was defined according to the rules described in the Methods. There were 92 patients (71.3%) in cluster 1, 109 patients (84.5%) in cluster 2, and 108 patients (86.4%) in cluster 3. Each cluster accounted for 20.0%, 17.1%, and 16.9% of the total variance, respectively, and Cronbach’s alpha values were 0.79, 0.72, and 0.82, respectively, which were considered acceptable. There were 4 patients with no symptom cluster, 14 patients with 1 cluster, 41 patients with 2 clusters, and 71 patients with 3 clusters.

Table 4 shows the results of a univariate or multivariate Cox proportional hazards regression analysis using the existence of symptom clusters and PPS as independent variables. Of the three clusters, cluster 2 was significantly (p = 0.018 and p = 0.023, respectively) associated with an increased risk of death in both the univariate and multivariate analyses. The hazard risks and their 95% confidence intervals from multivariable analysis revealed that those who had cluster 2 had a two-fold increased risk of death compared to patients who did not. In addition, poor PPS (≤ 30) was significantly associated with an increased risk of death.

**Table 4:** Results of Cox proportional hazards regression analysis of survival data

	Univariate analysis (n = 130)						Multivariate analysis (n = 117)				
Variables	n	Estimates	S.E	HR	95% CI	*p*	Estimates	S.E	HR	95% CI	*p*
PPS											
≥ 70	20	0	―	1	Reference		0	―	1	Reference	
40–60	74	0.329	0.290	1.389	0.787–2.452	0.257					
≤ 30	29	1.192	0.328	3.292	1.729–6.267	< .001**	0.986	0.246	2.679	1.653–4.343	< .001**
Cluster 1											
No	37	0	―	1	Reference						
Yes	92	0.007	0.222	1.007	0.652–1.557	.974					
Cluster 2											
No	20	0	―	1	Reference		0	―	1	Reference	
Yes	109	0.703	0.297	2.019	1.129–3.611	.018*	0.731	0.322	2.077	1.104–3.907	.023*
Cluster 3											
No	17	0	―	1	Reference						
Yes	108	0.324	0.290	1.382	0.784–2.438	.264					

*PPS* Palliative Performance Scale. Cluster 1: pain (pain and pain interference), insomnia, emotional functioning (felt tense and felt depressed). Cluster 2: dyspnea, appetite loss, fatigue (felt weak and tired), and nausea. Cluster 3: physical functioning (short walk, in bed, and need help)

^*^p < 0.05, **p < 0.01; *estimates* regression coefficients, *S.E.* standard errors of estimates, *HR (95% CI)* hazard ratio and 95% confidence interval

**Fig. 1:**
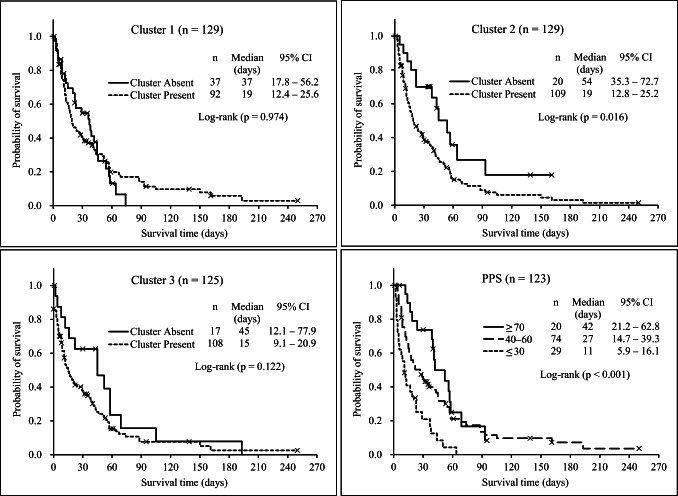
Relationships of the presence of clusters 1, 2, and 3 and Palliative Performance Scale (PPS) with survival. The inserted numbers represent the number of patients (n), median survival time in each group, and 95% confidence interval (CI). Cluster 1: pain (pain and pain interference), insomnia, and emotional functioning (felt tense and felt depressed). Cluster 2: dyspnea, appetite loss, fatigue (felt weak and been tired), and nausea. Cluster 3: physical functioning (short walk, in bed, and need help)

Figure 1 shows the Kaplan–Meier survival curves to evaluate the influence of the existence of each symptom cluster and PPS on survival, where the log-rank test indicated a significant difference in cluster 2 (p = 0.016) and PPS (p < 0.001). For cluster 1 and cluster 3, the survival curves were not significantly different regardless of whether these symptoms existed. However, the median survival time of each cluster was shorter in the presence of a cluster compared to its absence.

## Discussion

We performed an observational study to examine the existence of symptom clusters based on a patient-reported QOL assessment tool in terminally ill patients with cancer hospitalized in a palliative care unit. Three symptom clusters were identified as follows: cluster 1 (pain (pain and pain interference), insomnia, emotional functioning (felt tense and felt depressed)), cluster 2 (dyspnea, appetite loss, fatigue (felt weak and been tired), and nausea), and cluster 3 (physical functioning (short walk, in bed, and need help)).

It has been reported that there are several shared mechanisms underlying the symptoms identified in cluster 1. The relationship between insomnia and pain is indicated as a physiological domain that includes neurological, metabolic, and other biochemical causes. In addition, the psychological domain includes emotional and affective factors such as mood and psychiatric disturbances, which are caused by and respond to both insomnia and pain [18]. In other words, insomnia and altered pain responses are considered to lead to poorer emotional functioning, such as depression. Jiménez et al. identified a neuropsychological cluster of symptoms including insomnia, depression, and anxiety, which is one of the psychological domains in advanced cancer patients, and reported that those symptoms should be treated concomitantly [19]. Our study suggests that pain (Q5, Q12), insomnia (Q6), and emotional functioning (Q13, Q14) could be summarized as a pain—neuropsychological cluster in terminally ill patients with cancer.

The symptoms identified in cluster 2, except for dyspnea, are characteristic of patients with cancer cachexia [20, 21]. Several studies have reported that CRP levels gradually increase in response to chronic systemic inflammation associated with cancer cachexia in the terminal stage [22, 23]. Laird et al. reported that elevated CRP levels are associated with pain, anorexia, dyspnea, and fatigue in patients with cancer [24, 25]. In our study, most patients had high CRP levels (median: 3.9 mg/dL); therefore, we considered that dyspnea, appetite loss, and fatigue excluding pain would exist in the same cluster. The symptoms in this combined cluster are consistent with previous reports [26], and Cheung et al. identified a cluster that includes fatigue, dyspnea, drowsiness, nausea, and appetite by assessing ESAS in outpatients with advanced cancer. In addition, similar to previous studies [3, 14], our results indicated that nausea and appetite loss exist in the same cluster as a gastrointestinal symptom cluster. These results suggest that dyspnea (Q4), appetite loss (Q8), fatigue (Q7, Q11), and nausea (Q9) could be summarized as a dyspnea—fatigue—gastrointestinal cluster even in terminally ill patients with cancer.

To our knowledge, the present study is one of few to investigate the symptom clusters of several distress symptoms that focused on patients with terminal cancer. It is difficult to determine whether the symptom clusters identified in our study are consistent in other terminally ill patient populations. This is because symptom clusters have been reported to be affected by several study methods, such as the chosen symptom assessment tool, the prevalence of symptoms in the study population, and patient backgrounds [16]. We used the QLQ-C15-PAL and identified symptom clusters, and found that the prevalence of the identified symptom clusters (clusters 1–3) was 70% or more. In addition, the variance of each cluster was 16.9 to 20.0%, and there were little differences between clusters. Therefore, we consider that terminally ill patients with cancer hospitalized in a palliative care unit may have any of the three symptom clusters detected in our analysis. Further validation studies of symptom clusters in terminally ill patients with cancer are needed to develop more effective approaches to relieve distress symptoms.

We identified a relationship between survival and symptom clusters detected using PCA. Among the three symptom clusters, the presence of cluster 2 (dyspnea, appetite loss, fatigue, and nausea) was a significant prognostic indicator. Moreover, the presence of this symptom cluster provided an approximately twofold increased risk of mortality in patients with a short prognosis of less than 3 weeks. Individual symptoms of dyspnea, appetite loss, and fatigue have been reported to be independent predictors of survival in patients receiving palliative care [9, 27]. Therefore, we hypothesized that cancer patients with a combination of these distress symptoms would have a higher risk of death. Our results indicate that it is important to confirm not only the presence of individual symptoms but also the presence of symptom clusters for the prediction of survival in terminally ill cancer patients.

Similar to our findings, Tsai et al. reported the association of symptom clusters with the survival in terminally ill cancer patients admitted to the palliative care unit [28]. The study used their original assessment scale called “Symptom Reporting Form” and evaluated the 15 symptoms such as fatigue, appetite loss, pain, dyspnea, taste alteration, and dysphasia at the time of hospitalization. As results, they detected 5 symptom clusters, loss of energy, poor intake, autonomic dysfunction, aerodigestive impairment, and pain complex. In the present study, we used QLQ-C15-PAL and identified 3 clusters from the scale measurements of 2 functional domains and 7 symptom domains, and the relationship between the symptom clusters and survival time was evaluated. These studies evaluated the symptom clusters using different assessment scales and obtained similar results, suggesting that our findings revealed that QLQ-C15-PAL can be also useful to assess symptom clusters affected survival in terminally ill patients with cancer.

PPS is widely used as a physician-assessed performance status and is well-known as a prognostic marker in patients with advanced cancer [29]. Lee et al. reported that both physical functioning scores among QLQ-C15-PAL, namely patient-reported performance status and PPS, showed a highly significant relationship with survival [30]. Therefore, we used PPS as a factor related to prognosis in addition to symptom clusters based on QLQ-C15-PAL. In contrast, our results indicated that PPS was a significant prognostic indicator; however, physical functioning (cluster 3) was not. Consequently, our results suggest that physician-assessed performance status is a better prognostic factor than the patient-reported status in terminally ill patients with cancer hospitalized in a palliative care unit.

This study had some limitations. First, our study was conducted in a palliative care unit in a single hospital. Second, the sample size was not large enough to ensure generalizability. Third, we excluded patients who did not complete the QLQ-C15-PAL questionnaire when hospitalized in the palliative care unit. Thus, our findings may not apply to patients with cognitive impairments or lack of consciousness, resulting in selection bias and inaccurate characterization of actual symptom clusters for terminally ill patients with cancer. Fourth, the QLQ-C15-PAL also limited the evaluation to only 14 items; thus, the clusters identified in our study may be oversimplified compared with another symptom assessment tool or terminally ill patient population. Finally, this secondary analysis was performed to investigate symptom clusters only at the time of hospitalization, and we did not conduct a longitudinal analysis.

## Conclusion

We examined symptom clusters based on PROs in terminally ill patients with cancer hospitalized in a palliative care unit. Using PCA, three symptom clusters were detected based on QLQ-C15-PAL scores, and the presence of the dyspnea—fatigue—gastrointestinal cluster and poor PPS indicated poor prognosis. The findings suggest that symptom clusters should be carefully considered to manage the distress symptoms in combination and predict survival in terminally ill patients with cancer. These results are useful when clinicians make personal decisions for terminally ill patients with cancer.

## Data Availability

Not applicable.
